# Modern contraceptive prevalence and its predictors among non-refugee and refugee Somali women in Nairobi city, Kenya; a comparative view

**DOI:** 10.3389/fgwh.2024.1328612

**Published:** 2024-01-19

**Authors:** Eliphas Gitonga, Anastasia J. Gage

**Affiliations:** ^1^ School of Health Sciences, Kenyatta University, Nairobi, Kenya; ^2^ Department of International Health and Sustainable Development, School of Public Health and Tropical Medicine, Tulane University, New Orleans, LA, United States

**Keywords:** contraception, Somali refugee, women, reproductive health, crisis

## Abstract

**Introduction and methods:**

This study sought to determine the prevalence and predictors of modern contraceptive use among non-refugee and refugee Somali women in Nairobi City, Kenya. The analysis was based on 976 currently married Somali women aged 15–39 years (non-refugees; 523, refugees; 415) who were interviewed in a 2021 household survey conducted in Kamukunji, Embakasi, and Ruaraka sub-counties of Nairobi City. The analysis was stratified by refugee status and multivariable logistic regression were run to determine predictors of modern contraceptive use in each group.

**Results:**

The prevalence of modern contraceptives was 34% for the total sample and 43% and 24% for non-refugees and refugees, respectively. The main methods of contraception among non-refugees were injectables, implants, and daily pills, while refugees mainly used male condoms, implants, and injectables. Stratified multivariable analysis showed that residence in formal vs. informal settlements was associated with significantly higher odds of modern contraceptive use among non-refugees but significantly lower odds among refugees, after controlling for other factors. Interaction terms confirmed that the strength of the associations of these variables with the odds of modern contraceptive use varied significantly by refugee status.

**Conclusion:**

Use of modern methods of contraception was lower among non-refugee and refugee Somali women compared to the national average and refugee status moderated the association of some predictor variables with the odds of modern contraceptive use. To increase use of modern contraceptives in urban areas, it is recommended that the Ministry of Health, refugee agencies, and county governments engage with the Somali community and implement appropriate interventions to empower refugee women economically and promote their access to and use of voluntary contraception services as soon as they settle in urban areas.

## Introduction

At the end of 2022, there were around 108 million displaced people worldwide. This included 35 million refugees, mainly in low- and middle-income countries (1). Kenya is one of the countries with the largest refugee populations in Africa, hosting an estimated 600,000 registered refugees as of 2022. Somalia, whose modern contraceptive prevalence is one percent, is the largest contributor of refugees in Kenya and Nairobi City (2), the majority of whom are adolescents and women (3, 4).

Refugees within urban areas in Kenya are deemed to be present illegally and are not supported by health policies, including contraception (5), which increases their vulnerability to unwanted pregnancies, sexually transmitted infections, and sexual violence. Adolescent and young women in humanitarian settings tend to have increased exposure to early or forced marriage, child trafficking, and coerced sex. According to studies conducted among refugees in Europe, teenage pregnancies are more than eight times as common as in non-refugee populations (6). In Kenya, unmet need for contraception is high among women of Somali descent who constitute most of the refugees. The unmet need for contraception among Somali women (non-refugees) in counties predominantly occupied by the Somali community ranges from 11% to 17% (7), compared to 20% and 39% among Somali refugees in Norway and Uganda, respectively (2, 8). In the most recent Kenya Demographic and Health Survey, the prevalence of modern contraceptive use was lowest in Wajir, Mandera, and Garissa counties, which are predominantly occupied by the Somali communities, and have total fertility rates ranging from 5.3 to 7.7 (7, 8).

High unmet need is associated with unintended pregnancies, maternal mortality, and unsafe abortions (9). The benefits of contraception include improved birth spacing, reduction of unwanted pregnancies, prevention of unsafe abortions, and improved maternal and child health. In a broader sense, contraception empowers women and ensures sustainable development in communities and countries (10). Thirty-five percent of maternal deaths can be prevented via modern contraceptive use (2).

Majority of Somali refugees do not use contraceptives because of varied barriers (11). Early marriage and high fertility have been persistent practices among Somali populations admitted to Kenyan refugee camps. Low contraceptive prevalence has been explained by the desire for large families to bring extra income and provide household assistance and caregiving (2). Refugees' low use of contraception has also been attributed to the limited availability of services in the areas where they live, loss of social support networks, and low priority assigned to contraceptive use in crisis response. Refugee women's vulnerability is aggravated by violence, conflict, poverty (12), and government policy. Refugees are not eligible for government health initiatives like national health insurance coverage (13, 14). Furthermore, Kenya does not have a policy for refugees to acquire citizenship.

Identifying the reproductive health needs of refugees is a global priority (15). Prior studies have indicated inter- and intra-group and community inequalities and disparities. Few studies have focused on disparities in contraceptive use between refugees and host communities (3). Most studies on refugee populations have also focused on those within camps and neglected those residing in urban areas (16). Data on modern contraceptive prevalence among urban refugees is limited. The present study sought to address these gaps in the literature by examining the prevalence and predictors of modern contraceptive use among non-refugee and refugee Somali women in Nairobi City. A critical focus was to investigate whether refugee status moderated the associations between commonly examined social determinants of health and modern contraceptive use. The data from this research will inform contraception policy and programming, especially among urban refugees.

## Methods

### Study design

The source of data is a 2021 urban cross-sectional household survey conducted in Kamukunji, Embakasi, and Ruaraka sub-counties of Nairobi City County. These sub-countries were selected because they host the highest concentration of Somalis in the city. The estimated sample size was 976 using United Nations guidelines (17) and assuming 95% confidence interval, a non-response rate of 10%, average household size of 6, and modern contraceptive prevalence of 60%.

Thirty clusters, each with roughly 100 households, were selected and acquired from Kenya's Bureau of Statistics. A household listing was conducted in each cluster by research assistants, accompanied by community health volunteers. Listing details included location, address, structure serial number, name of the head of household, and usual number of members in the household. Thirty Somali women were interviewed in each cluster. In households with more than one eligible woman, random sampling was used to select one to participate in the study.

### Study population

The study population was made up of non-refugee and refugee married Somali women aged 15–39 years. The inclusion criteria were all consenting married Somali women living in the study area, aged 15–39 years. Women who were critically or mentally ill were excluded. To determine refugee status, a direct question (“Are you a refugee?”) and proxy indicators (inclusion in free maternity health care (reserved only for Kenyan citizens); use of the national health insurance fund (given only to Kenyan citizens with identification); and previous town or place of residence (residence in Ethiopia and Somalia) were used.

### Data collection

The questionnaires were interviewer administered. Kobo Collect mobile phone data capture software was used for data collection. The research assistants were graduates and could communicate with Somali women. Training encompassed community entry, interview skills, mobile data capture and transmission, and ethical guidelines. Supervision was done daily during the entire period of data collection to ensure quality. Pretesting of the tool was done in parts of Ruaraka Sub County among 100 participants, after which corrections were made for final data collection. The data collection was done between February and December 2021.

### Ethical and logistical approval

Ethical approval was granted by the Mount Kenya University review committee (MKU/ERC/1619). Every participant consented to being interviewed within ethical principles and guidelines. The research permit was granted by the National Commission for Science, Technology, and Innovation (884,237). Nairobi Metropolitan Services issued permission to proceed with the study (EOP/NMS/HS/7/031). Community entry also included linkage and continuous communication with the sub-county reproductive health coordinator. During data collection, a Somali community health volunteer accompanied the research assistants to the households for acceptability and data capture. In some circumstances, a community elder was involved to improve community acceptability.

### Variables

The dependent variable was dichotomous and measured current use of a modern method of contraception. Modern methods include male and female sterilization, injectables, intrauterine devices (IUDs), contraceptive pills, implants, female and male condoms, emergency contraception, the Standard Days Method, and the lactational amenorrhea method. According to USAID, the lactational amenorrhea method and the standard days method are modern contraceptive methods because they are safe, effective at preventing pregnancy, require sound understanding of biology, include protocols for proper use, and have undergone appropriate testing to determine their efficacy under various conditions (18). Additionally, only 1.3% and 3.9%, respectively, of non-refugees and refugees, in the current study used them.

The independent variables included age group (15–24, 25–29, 30–39), type of settlement (formal or informal), highest level of education attended (no formal, primary, secondary, and tertiary), main source of income [none including those on relief support, casual work, salaried employment, and business (i.e., micro-business); later salary and micro-business were combined for binary regression], and duration of residence in Nairobi in years (0–9, 10–19, 20–29, and 30 and more; later they were grouped as less than 10 years and more than 10 years for binary regression),, disability status (living or not living with disability). The UN definition of informal settlements was used in this study: high population density and lacking one or more of the following conditions: access to improved water, access to improved sanitation, sufficient living area, housing durability, and security of tenure (19). The businesses run by women in the study area fell in the micro enterprises category, that is, less than 10 employees and an annual turnover of USD 7500 (20).

### Statistical analysis

The data were entered into STATA version 16 for cleaning and analysis. Descriptive statistics and Chi-Square tests were used to determine the statistical significance of differences in socioeconomic characteristics and modern contraceptive prevalence between non-refugee and refugee women. Multivariable logistic regression was used to identify predictors of current use of a modern contraceptive method and the results were expressed as adjusted odds ratios and 95% confidence intervals.

The data were examined for missing values. Overall, 38 participants had missing values on one or more variables of interest. There were no statistically significant differences between the women with and those without missing values as indicated by the *p*-values (education: *p* = 0.73; age: *p* = 0.94; settlement: *p* = 0.22; disability: *p* = 0.48; and modern contraceptive use: *p* = 0.17). The analysis was restricted to women with no missing values on any of the variables included in the analysis, resulting in an analytic sample of 938. Multicollinearity was examined and the mean variance inflation factor was established to be 1.04, which indicated that multicollinearity was not of concern.

## Results

### Sample characteristics

Table 1 shows the socio-demographic characteristics of participants by refugee status. There were significant differences in the age distribution, educational level, type of place of residence, main source of income, and duration of residence in Nairobi between non-refugee and refugee women (*p* < 0.001). Non-refugee women were on average two and a half years older than refugee women (mean age: 28.7 and 26.1 years, respectively) and had higher levels of education. About two in five non-refugees had attended secondary or higher education, compared to 28% of refugees. Most non-refugees resided in informal settlements, while refugees mainly lived in formal settlements. While non-refugees and refugees had similar employment rates, a third of non-refugees derived their income primarily from microbusinesses, compared to slightly more than half of refugees (34% vs. 55%). About three percent of participants lived with a physical disability. Disability status did not vary significantly by refugee status (*p* = 0.131). Significantly fewer non-refugees than refugees had lived in Nairobi for less than 10 years (47% vs. 56%).

**Table 1:** Percent distribution of currently married Somali women by background characteristics and refugee status, Nairobi, 2021.

Variables	Categories	Total sample (*n* = 938)		Non-Refugees (*n* = 523)		Refugees (*n* = 415)		*p*-values
		Number	Percent (%)	Number	Percent (%)	Number	Percent (%)	
Age	15–24	328	36.0	147	28..1	181	43.6	<0.001
	25–29	244	26.0	150	28.7	94	22.7	
	30–39	366	39.0	226	43.2	140	33.7	
	Mean	27.5		28.7		26.1		<0.001
Education	No formal	245	26.1	118	22.6	127	30.5	<0.001
	Primary	343	36.6	171	32.7	172	41.5	
	Secondary/higher	350	37.1	234	44.7	116	28.0	
Settlement	Informal	473	50.4	353	67.5	120	28.9	<0.001
	Formal	465	49.6	170	32.5	295	71.1	
Main source of Income	None	148	15.8	91	17.4	57	13.7	
	Casual work	316	33.7	213	40.7	103	24.8	
	Microbusiness	408	43.5	180	34.4	228	54.9	
	Salary	66	7.0	39	7.5	27	6.5	
Disability status	No	909	96.9	509	97.3	400	96.4	0.131
	Yes	29	3.1	14	2.7	15	3.6	
Duration lived in the city in years	0–9	478	51.0	244	46.5	234	56.4	<0.001
	10–19	220	23.4	106	20.3	114	27.5	
	20–29	165	17.6	114	21.8	51	12.3	
	30 and above	75	8.0	59	11.3	16	3.9	

### Contraceptive prevalence and method mix

Table 2 shows the contraceptive prevalence rate for non-refugee and refugee Somali women. One out of three women in the sample was using a method of contraception at the time of the survey (any method: 38%; modern method: 34%). Non-refugees had significantly higher contraceptive use rates than refugees. For example, the modern contraceptive prevalence rate was 43% and 24% among non-refugees and refugees, respectively (*p* < 0.001). Method mix varied by refugee status, with refugee women reporting more male condom use than non-refugee women. Among non-refugees, the most common contraceptive methods were injectables (37%) followed by implants (24%) and pills (20%). Among refugees, the dominant method was the implant (28%), followed by male condom (26%), and injectable (18%). The *χ*^2^ statistics showed that the difference in method mix between non-refugees and refugees were statistically significant (*p* < 0.001). About one in three women in either group used long-acting reversible contraceptives. In both groups, female condoms, bilateral tubal ligation, vasectomy, lactational amenorrhea methods, and emergency contraception accounted for less than 5% of method share. Traditional methods were relatively unimportant. Rhythm and withdrawal accounted for 3% and 4%, respectively, among non-refugees and 1% each among refugees.

**Table 2:** Percent distribution of currently married women by contraceptive method and modern contraceptive prevalence according to refugee status of Somali women, Nairobi, 2021.

Variable	Category	Total sample (*n* = 938)		Non-refugees (*n* = 523)		Refugees (*n* = 415)		*p*-values
		Number	Percent	Number	Percent	Number	Percent	
Any Method	Current use	344	36.7	242	46.3	102	24.6	<0.001
Modern Methods	Current use	321	34.2	223	42.6	98	23.6	<0.001
Method Type^a^	Injectable	107	31.1	89	36.8	18	17.7	<0.001
	Pill	56	16.3	48	19.8	8	7.8	
	Implant	87	25.3	58	24.0	29	28.4	
	Intra-uterine device	22	6.4	12	5.0	10	9.8	
	Male condom	33	9.6	7	2.9	26	25.5	
	Female condom	1	0.3	0	0.0	1	1.0	
	Bilateral tubal ligation	3	0.9	1	0.4	2	2.0	
	Standard Days Method	5	1.5	4	1.8	1	1.0	
	Vasectomy	0	0.0	0	0.0	0	0	
	Lactational amenorrhea method	7	2.0	3	1.2	4	3.9	
	Emergency contraception	3	0.9	2	0.8	1	1.0	
	Rhythm	9	2.6	8	3.3	1	1.0	
	Withdrawal	11	3.2	10	4.1	1	1.0	

^a^ Data pertain to 344 currently married women who were using a method of contraception at the time of the survey.

### Bivariate analysis

Table 3 shows socioeconomic differentials in the use of modern contraceptive methods by refugee status. The prevalence of modern contraceptive use among non-refugee women was highest (45%–64%) among the following subgroups: those aged 30–39 years, with no formal education, living within formal settlements, salaried, living with a disability, and residing in the city for at least 20 years. Among non-refugee women, significant differentials in modern contraceptive use occurred by type of settlement (*p* < 0.030) and main source of income (*p* = 0.025. For example, 49% of non-refugee women living in formal settlements were currently using a modern method compared to 39% of their counterparts living in formal settlements. Among refugee women, the use of modern contraceptive methods was proportionally higher among women with the following characteristics: age 30–39 (34%), attended secondary or higher education (31%), lived in informal settlements (38%), had no source of income or were beneficiaries of relief support (35%), were living with disabilities (60%), and had lived in the city for 30 years or more (38%). Among refugee women, the modern contraceptive prevalence rate varied significantly by age (*p* < 0.001), type of settlement (*p* < 0.001), disability status (*p* < 0.001), and duration of residence in the city (*p* = 0.009).

**Table 3:** Percentage of currently married Somali women who were currently using a modern method of contraception, by background characteristics and refugee status, Nairobi, 2021.

Variables	Categories	Non refugee (*n* = 523)			Refuge (*n* = 415)		
		Number	Modern contraceptive use (%)	*p*-value	Number	Modern contraceptive use (%)	*p*-value
Age	15–24	147	42.9	0.462	181	12.7	<0.001
	25–29	150	38.7		94	29.8	
	30–39	226	45.1		140	33.6	
Education	No formal	118	47.5	0.426	127	22.8	0.065
	Primary	171	39.8		172	19.2	
	Secondary/higher	234	42.3		116	31.0	
Type of settlement	Informal	353	39.4	0.030	120	37.5	<0.001
	Formal	170	49.4		295	18.0	
Main source of Income	None	91	41.8	0.025	57	35.1	0.184
	Casual work	213	38.0		103	21.4	
	Microbusiness	180	43.9		288	21.9	
	Salary	39	64.1		27	22.2	
Disability	No	509	42.4	0.572	400	22.3	<0.001
	Yes	14	(50.0)		15	(60.0)	
Duration lived in the city in years	0–9	244	40.2	0.387	234	17.5	0.009
	10–19	106	39.6		114	29.8	
	20–29	114	48.3		51	33.3	
	30 and above	59	47.5		16	(37.5)	

Estimates in parentheses are based on less than 25 cases.

Entries in the column headed “Number” pertain to the denominators on which the calculations of the modern contraceptive prevalence rate are based. Numerators can be derived by multiplying the modern contraceptive prevalence rate and the denominator.

### Multivariable analysis

Table 4 shows adjusted odds ratios (AOR) and 95% CI from multivariable logit models of modern contraceptive use. The analysis was stratified by refugee status. Among non-refugee women, the AOR of modern contraceptive use was 1.7 times higher among those living in formal vs. informal settlements. Among refugee women, the only significant predictors of modern contraceptive use were age and the type of settlement. While settlement in formal settlements was associated with significantly higher odds of modern contraceptive use than residence in informal settlements (AOR = 1.67; 95% CI = 1.12–2.46; *p* = 0.010) among non-refugee women, the opposite result was found among refugee women (AOR = 0.29; 95% CI = 0.17, 0.5; *p* < 0.001). In addition, refugee women aged 25–29 years (AOR = 3.3, 95% CI = 1.67–6.40); *p* = 0.001) and 30–39 years (AOR = 3.6, 95% CI = 1.89–6.83, *p* < 0.001) years had higher odds of modern contraceptive use. Regardless of refugee status, education, main source of income, disability status and duration of stay in the city were not significant predictors of modern contraceptive use.

**Table 4:** Results of multivariable logistic regression models of current use of modern contraception among currently married Somali women, by refugee status, Nairobi, 2021.

Variables		Non-refugees (*n* = 523)		Refugees (*n* = 415)	
		AOR (95% CI)	*p*-value	AOR (95% CI)	*p*-value
Age	15–24	Reference		Reference	
	25–29	0.82 (0.51–1.31)	0.405	3.26 (1.67–6.40)	0.001
	30–39	0.99 (0.63–1.54)	0.973	3.60 (1.89–6.83)	<0.001
Education	No formal	Reference		Reference	
	Primary	0.65 (0.39–1.05)	0.084	1.11 (0.60–2.04)	0.752
	Secondary/higher	0.69 (0.43–1.13)	0.144	1.83 (0.94–3.55)	0.074
Settlement	Informal	Reference		Reference	
	Formal	1.67 (1.12–2.46)	0.010	0.29 (0.17–0.50)	<0.001
Main source of Income	None/relief support	Reference		Reference	
	Casual jobs	0.82 (0.49–1.37)	0.469	0.76 (0.34–1.72)	0.509
	Microbusiness/salary	1.26 (0.76–2.09)	0.368	0.82 (0.41–1.66)	0.600
Disability status	No	Reference		Reference	
	Yes	1.63 (0.55–4.82)	0.369	2.66 (0.753–9.46)	0.128
Duration lived in the city	Less than 10 years	Reference		Reference	
	More than 10 years	1.213 (0.844–1.74)	0.0.295	1.64 (0.96–2.80)	0.069
Constant		0.78 (0.41–1.46)	0.441	0.23 (0.09–0.58)	0.002
Log 2 Log		−349.20		−196.91	
*N*		523		415	

To examine whether refugee status moderated the association between some of our independent variables and modern contraceptive use, we combined the refugee and non-refugee samples and added interaction terms between: (1) refugee status and education; (2) refugee status and formal settlement; and (3) refugee status and main source of income. The purpose of testing for interaction terms was to ascertain whether the association of education, type of settlement, and main source of income with modern contraceptive use varied significantly between refugee and non-refugee women, after controlling for other factors.

Table 5 presents the results of our regression models with interaction terms. Residence in formal vs. informal settlements was associated with significantly lower odds of modern contraceptive use among refugees than among non-refugees (interaction term: AOR = 0.207; 95% CI = 0.109–0.393; *p* < 0.001). Salaried employment (vs. unemployment/relief support) was also associated with significantly lower odds of modern contraceptive use among refugees than non-refugees (Interaction term: AOR = 0.238; 95% CI = 0.061–0.934; *p* = 0.040). However, secondary/higher education (vs. no formal education) was associated with significantly higher odds of modern contraceptive use among refugees compared to non-refugees (interaction term: AOR = 2.316; 95% CI = 1.071–5.011; *p* = 0.033).

**Table 5:** Results of multivariable logistic regression models of current use of modern contraception among currently married Somali women, with interaction terms between refugee status and selected variables, Nairobi, 2021.

Variables	AOR	95% CI	*p*-value
Age			
15–24	Reference		
25–29	1.373	0.938–2.010	0.102
30–39	1.593	1.1.114–2.276	0.011
Education			
No formal	Reference		
Primary	0.662	0.406–1.081	0.099
Secondary/higher	0.745	0.461–1.203	0.230
Settlement			
Informal	Reference		
Formal	1.638	1.108–2.4121	0.013
Main source of income			
None/relief support	Reference		
Casual jobs	0.828	0.489–1.383	0.472
Microbusiness/Salary	1.271	0.765–2.114	0.354
Disability status			
No	Reference		
Yes	2.154	0.973–4.769	0.058
Duration lived in the city (years)			
Less than 10 years	Reference		
More than 10 years	1.418	1.054–1.909	0.021
Refugee status			
Non-refugee	Reference		
Refugee	1.018	0.414–2.502	0.969
Interaction terms			
Settlement			
Refugee # formal	0.211	0.112–0.399	<0.001
Education			
Refugee # primary	1.506	0.701–3.236	0.293
Refugee # secondary/higher	2.24	1.0744–4.820	0.038
Main source of income			
Refugee # casual	0.753	0.299–1.892	0.546
Refugee # microbusiness/salary	0.525	0.227–1.213	0.132
Constant	0.483	0.267–0.871	0.016
−2 Log Likelihood	−554.871		
*N*	938		

The model is based on the total sample of 938.

## Discussion

This study sought to establish the prevalence and predictors of modern contraceptive use among refugee and non-refugee Somali women. It further sought to determine whether refugee status moderated the association between selected predictor variables and modern contraceptive use. The prevalence of modern contraceptives among the currently married Somali women surveyed was 34%. Non-refugees had considerably higher modern contraceptive prevalence rates than refugees (43% vs. 24%), but in both groups, the.

Prevalence rates were lower than the national average (57%) and the rate for Nairobi City (56%). However, the rates were higher than in some of the north eastern counties with high concentrations of Somali, notably Garissa, Mandera, and Wajir, where the percentage of currently married women who were using a modern method of contraception ranged from 2% to 11% in the 2022 Kenya Demographic and Health Survey (7). The modern contraceptive prevalence rate among urban Somali refugees in our sample was lower than the prevalence found in refugee camps in Rwanda which was 32%–40% (21). It was however higher than levels found in refugee camps in the Democratic Republic of the Congo, Sudan, and Uganda, which ranged from 1.7% in South Darfur to 16.2% in Northern Uganda (40%) (22).

The most common methods of contraception among non-refugees in our sample were injectables, implants, and the pill, while refugees mainly used male condoms, implants, and injectables. The use of long-term contraceptives was 29% and 35% among non-refugees and refugees respectively. This demonstrated that both non-refugees and refugees mainly used short-term methods, except for implants. Their method mix was comparable to that of refugees in camps in several countries (Rwanda, Cox's Bazar, Bangladesh; Ali Addeh, Djibouti; Amman, Jordan; Eastleigh, Kenya; Kuala Lumpur, Malaysia; and Nakivale, Uganda) whose dominant methods were short-term methods like pills and injectables (21, 23), and to Somali refugees in Kampala (8). However, the method mix among Somali refugees in our sample differed from that of Palestinian refugees, whose dominant methods were intrauterine devices (24). This may be related to preferences for specific contraceptive methods among support organizations and refugee women, and to cultural factors.

Our stratified multivariable analysis showed that residence in formal vs. informal settlements was associated with significantly higher odds of modern contraceptive use among non-refugees but significantly lower odds among refugees, after controlling for other factors. Interaction terms confirmed that the associations of these variables with the odds of modern contraceptive use varied significantly by refugee status. The findings for non-refugees were consistent with other studies (25) and may be attributed to better access to contraception and reproductive health services and economic status. However, the findings for refugees on higher odds of modern contraceptive use within slums were unexpected. Studies in Kenya and Democratic Republic of the Congo have found similar results among women living in slum vs. non-slum settlements (26, 27). Our results may be partly explained by the efforts of multiple players that support refugees' reproductive health, including civil society which supplies contraceptive services within informal settlements among the refugees. These multiple players cushion the refugees from barriers imposed by the government on urban refugees.

Another unexpected finding was that refugee status moderated the association between education and modern contraceptive use. Secondary/higher education vs. no education was associated with significantly higher odds of modern contraceptive use among refugees than non-refugees, even after controlling for other factors, including duration of residence in Nairobi City. It is possible that among non-refugees, there is greater cultural cohesion, which could lead to the maintenance or reinforcement of social norms that are supportive of childbearing and non-use of modern contraception. These factors may reduce the potential effects of secondary/higher education on modern contraceptive use among non-refugees. By comparison, the economic, social, and legal uncertainties associated with refugee status may strengthen the association between secondary/higher education and contraception among refugee Somali women, even if they tend to reside with non-refugee Somalis who share similar cultural beliefs. In addition, due to refugee status, the husbands/partners are sometimes absent (mainly due to separation that occurs during conflict including remaining in the fragile country, death and relocation) which may result in women having greater power in decision making about contraceptive use (8). It will be important for future research to shed light on pathways/mechanisms underlying the moderating effect of refugee status on common social determinants of modern contraceptive use.

### Knowledge gaps filled by this study

This research has demonstrated differences in the prevalence and predictors of modern contraceptive use between non-refugee and refugee Somali women living in a major urban area. Despite the increase in conflicts in sub-Saharan Africa, there has been limited research on refugees in urban settings and comparisons of their contraceptive behavior with that of non-refugees. This understanding is critical for targeted interventions in upscaling modern contraception among urban refugees.

### Limitations

The cross-sectional survey design is a limitation of this study as causality could not be established. The study also focused on married women because of Somali culture and for community entry, as most Somali opinion leaders and gatekeepers do not condone sex outside marriage. This limited our understanding of contraceptive behavior among unmarried, sexually active Somali women. Finally, the study was conducted during the active COVID-19 period, which limited some human interactions and affected economic activities within the area of study. The sample is also not representative of Somali refugees and non-refugees in Kenya. Due to lack of data, our analysis does not control for some individual characteristics such as women's role in decision making about contraceptive use, exposure to contraception messages, and perceived social norms; husband's characteristics; community characteristics; and aspects of the supply environment, such as access to family planning services and quality of care, which have been found to influence women's use of modern contraception.

## Conclusion and recommendations

The use of modern contraception is low among both refugee and non-refugee Somali women compared to the national average. The predictors of the use of modern contraceptives among non-refugee Somali women are the main source of income and type of settlement. Among refugee Somali women, predictors of contraceptive use are type of settlement and duration of residence in the city. Refugee status moderated the association of type of settlement, main source of income, and education with modern contraceptive use. To increase use of modern contraceptives in urban areas, the Ministry of Health, refugee agencies, and county governments who engage with the Somali community should put in place appropriate interventions to ensure refugees are able to access and use modern contraceptives within a shorter duration after migrating to the city. Future research should examine the role of factors that were omitted in our study and examine the pathways through which refugee status influences Somali women's use of modern contraception.

## Data Availability

The raw data supporting the conclusions of this article will be made available by the authors, without undue reservation.

## References

[1] UNHCR. Global Trends: Forced Displacement in 2022. Copenhagen, Denmark: UNHCR (2023).

[2] Gele AA Shrestha M Sheikh NS Qureshi SA. Pregnant and powerless: exploring barriers to contraceptive use among women in Mogadishu, Somalia. Health Serv Res Manag Epidemiol. (2022) 9:233339282211170. 10.1177/2333392822111705

[3] Singh NS Prabhakar P Ssali A Namakula S Namatovu J Kapiti R “They will say you want to make their home die”: a mixed methods study to assess modern family planning use in partnered south Sudanese refugee and host populations in northern Uganda. PLoS Glob Public Health. (2022) 2(6):e0000348. 10.1371/journal.pgph.0000348 36962421 PMC10022387

[4] Raben LAD van den Muijsenbergh METC. Inequity in contraceptive care between refugees and other migrant women?: a retrospective study in Dutch general practice. Fam Pract. (2018) 35(4):468–74. 10.1093/fampra/cmx133 29351609

[5] Figures at a Glance - UNHCR Kenya. UNHCR; UNHCR. (2019). Available online at: https://www.unhcr.org/ke/figures-at-a-glance (Accessed June 15, 2023).

[6] Kativa M Blessing M. Urban refugees in Nairobi: tackling barriers to accessing housing, services and infrastructure. (2019). Available online at: https://www.environmentandurbanization.org (Accessed June 15, 2023).

[7] KNBS and ICF. Kenya Demographic and Health Survey 2022: volume 1. Nairobi, Kenya, and Rockville, Maryland, USA: KNBS and ICF (2023).

[8] Abdulahi M Kakaire O Namusoke F. Determinants of modern contraceptive use among married Somali women living in Kampala; a cross sectional survey. Reprod Health. (2020) 17(1):72. 10.1186/s12978-020-00922-x 32448285 PMC7247175

[9] Rattan J Noznesky E Curry DW Galavotti C Hwang S Rodriguez M. Rapid contraceptive uptake and changing method mix with high use of long-acting reversible contraceptives in crisis-affected populations in Chad and the democratic republic of the Congo. Glob Health Sci Pract. (2016) 4(Suppl 2):S5–20. 10.9745/GHSP-D-15-00315 27540125 PMC4990162

[10] Boadu I. Coverage and determinants of modern contraceptive use in sub-saharan Africa: further analysis of demographic and health surveys. Reprod Health. (2022) 19(1):18. 10.1186/s12978-022-01332-x 35062968 PMC8781110

[11] Kiura AW. Constrained agency on contraceptive use among Somali refugee women in the kakuma refugee camp in Kenya. Gend Technol Dev. (2014) 18(1):147–61. 10.1177/0971852413515321

[12] West L Isotta-Day H Ba-Break M Morgan R. Factors in use of family planning services by Syrian women in a refugee camp in Jordan. J Fam Plann Reprod Health Care. (2016) 43(2):96–102. 10.1136/jfprhc-2014-101026 26962045

[13] International Institute for Environment and Development. International Institute for Environment and Development (n.d).

[14] Jemutai J Muraya K Chi PC Mulupi S. A situation analysis of access to refugee health services in Kenya: gaps and recommendations - A literature review. Working papers 178cherp, centre for health economics. University of York (2021).

[15] Nara R Banura A Foster AM. Assessing the availability and accessibility of emergency contraceptive pills in Uganda: a multi-methods study with Congolese refugees. Contraception. (2020) 101(2):112–6. 10.1016/j.contraception.2019.09.008 31655072

[16] Earle L. “A World without Refugee Camps? IIED Launches Research on Urban Refugees.” *International Institute for Environment and Development*, Available online at: www.iied.org/world-without-refugee-camps-iied-launches-research-urban-refugees (Accessed October 24, 2023).

[17] Designing Household Survey Samples: Practical Guidelines Logo. (2005). Available online at: https://unstats.un.org/unsd/demographic/sources/surveys/Handbook23June05.pdf (Accessed June 20, 2023).

[18] Malarcher S Spieler J Fabic MS Jordan S Starbird EH Kenon C. Fertility awareness methods: distinctive modern contraceptives. Glob Health Sci Pract. (2016) 4(1):13–5. 10.9745/GHSP-D-15-00297 27016540 PMC4807745

[19] UN-HABITAT. World Cities Report 2016; Urbanization And Development - Emerging Futures. Nairobi, Kenya: UN-Habitat (2016).

[20] Eliud Moyi. *Developing a Marketing Framework for Micro and Small Enterprises in Kenya* (2006).

[21] Nyirimanzi A Butare B Iryanyawera MC Mutamba D Mugisha M Kayumba PC. The utilisation of modern contraceptives in refugee camps. Rw Public Health Bul. (2020) 2(3):15–8.

[22] McGinn T Austin J Anfinson K Amsalu R Casey SE Fadulalmula SI Family planning in conflict: results of cross-sectional baseline surveys in three African countries. Confl Health. (2011) 5:11. 10.1186/1752-1505-5-11 21752241 PMC3162885

[23] Tanabe M Myers A Bhandari P Cornier N Doraiswamy S Krause S. Family planning in refugee settings: findings and actions from a multi-country study. Confl Health. (2017) 11:9. 10.1186/s13031-017-0112-2

[24] Hababeh M Zeidan W El-Kader MA Al Thaher A Kassim N Habash E Contraceptive use by palestine refugee mothers of young children attending UNRWA clinics: a cross-sectional follow-up study. Lancet. (2018) 391:S26. 10.1016/s0140-6736(18)30392-1 29553424

[25] Speizer IS Nanda P Achyut P Pillai G Guilkey DK. Family planning use among urban poor women from six cities of uttar pradesh, India. J Urban Health. (2012) 89(4):639–58. 10.1007/s11524-011-9667-1 22399250 PMC3535138

[26] Duminy J Cleland J Harpham T Montgomery MR Parnell S Speizer IS. Urban family planning in low- and middle-income countries: a critical scoping review. Glob Womens Health. (2021) 2:749636. 10.3389/fgwh.2021.749636 PMC8593933 34816250

[27] Akilimali PZ Tran N-T Gage AJ. Heterogeneity of modern contraceptive use among urban slum and nonslum women in Kinshasa, DR Congo: cross-sectional analysis. Int J Environ Res Public Health. (2021) 18(17):9400. 10.3390/ijerph18179400 34502009 PMC8430884

